# Roles undertaken by family caregivers to help individuals with cancer manage symptoms: a qualitative study of caregivers, patients, and oncology clinicians

**DOI:** 10.1007/s00520-026-10500-9

**Published:** 2026-02-27

**Authors:** J. Nicholas Odom, Nicole L. Henderson, Tanvi V. Padalkar, Reed W. R. Bratches, Macy L. Stockdill, Etzael Ortiz Olguin, Gabrielle B. Rocque

**Affiliations:** 1 School of Nursing, University of Alabama at Birmingham, 1720 2nd Avenue South, Birmingham, AL 35294 USA; 2 Department of Medicine, University of Alabama at Birmingham, 1720 2nd Avenue South, Birmingham, AL 35294 USA; 3 Department of Medicine, University of Alabama at Birmingham, 1808 7th Avenue South, Birmingham, AL 35233 USA; 4 Department of Medicine, University of Alabama at Birmingham, 1824 6th Ave South, Birmingham, AL 35924-3300 USA

**Keywords:** Family caregiver, Caregiving, Cancer, Symptom management

## Abstract

**Purpose:**

Identify and describe the distinct roles family caregivers undertake to help patients with cancer manage symptoms from diverse constituency perspectives.

**Methods:**

Qualitative study employing one-on-one, semi-structured interviews with family caregivers (*n* = 20), their care recipients with stage I–IV cancers (*n* = 20), and oncology clinicians involved with remote symptom monitoring program (*n* = 25). Participants were recruited from a large academic comprehensive cancer center between January 2025 and July 2025. Interviews were professionally transcribed and analyzed using a thematic analysis approach.

**Results:**

Caregivers averaged 59 years, were 60% White and 40% Black race, and were mostly female (70%) and the patient’s spouse/partner (40%). Patients averaged 58 years and were mostly female (75%) with a wide range of cancer types. Clinicians averaged 37 years and included nurses (32%), physicians (24%), and non-clinical navigators (20%). Themes included (1) assisting with hands-on and direct symptom management; (2) providing emotional support and motivation; (3) promoting preventative self-care and restorative behaviors; and (4) serving as symptom reporter, advocate, and information seeker. Clinicians primarily stressed the role caregivers played as a symptom reporter and advocate. (5) A fifth theme we noted was the challenge caregivers have in finding the right balance of helping while respecting patients’ autonomy and independence.

**Conclusion:**

These findings elucidate the symptom partnering roles that cancer family caregivers play in how patients manage their symptoms and the key struggles in navigating this role. Optimizing these roles represents novel targets for interventions to support the symptom partnering role of family caregivers.

## Introduction

Family caregivers of individuals with cancer often assume an influential role in helping their care recipients manage the physical and psychological symptoms resulting from cancer and its often-intensive treatment regimens [1]. Physically, patients can experience pain, fatigue, nausea and vomiting, appetite loss, impaired sleep, and neuropathy [2]. Psychologically, patients can experience anxiety, depressed mood, loneliness, cognitive slowness and memory difficulties (sometimes called “chemo brain”), and existential distress [3]. These symptoms are not only prevalent, they can also intensify across the cancer trajectory from diagnosis to treatment to survivorship to end of life [4, 5]. Furthermore, they can reduce functional capacity and the ability of patients to undertake normal activities of day-to-day living [3].

Consequently, most individuals with cancer rely on the assistance of family caregivers to not only directly assist with symptom management but also to compensate for the functional limitations imposed by symptom-related impairments [1]. A population-based US survey by the National Alliance for Caregiving [6] reported that 61% of cancer family caregivers assist with giving medicines, pills, or injections. A quarter (24%) deal with incontinence/diapers and over half (57%) help their care recipient get in and out of beds and chairs. While such assistance can support symptom control, the mounting number of responsibilities to caregivers raises concerns about caregiver burden, preparedness, and patient safety risks. This reliance on caregiver assistance may become even more pronounced with the growing trend of chemotherapy being delivered in the home setting, placing additional responsibilities on cancer caregivers [7, 8].

The symptom management partnership between patients and caregivers in oncology has been broadly acknowledged particularly through qualitative work describing caregivers’ involvement in monitoring, administering medication and providing psychosocial support [9, 10]. However, prior work has often not been directly focused on the symptom support roles of caregivers and has been almost exclusively limited to patient or caregiver reports [9]. Notably, there is an absence of studies that have included the additional perspective of oncology clinicians who not only primarily oversee and medically manage patients’ symptoms but who also interact with and are in a position to support caregivers in their roles. Hence, combining and triangulating the diverse perspectives of caregivers, patients, and clinicians potentially offers a more holistic and multi-dimensional understanding of symptom management in cancer. Thus, our study aimed to identify and describe how caregivers help patients manage symptoms and to furthermore contrast perspectives between family caregivers, patients, and oncology clinicians.

## Methods

This was a qualitative descriptive study conducted as part of a larger set of summative and formative evaluation studies to investigate intervention and implementation approaches to optimizing the role of patients, clinicians, and family caregivers in remote patient symptom monitoring [11–15]. One-on-one, digitally recorded, semi-structured interviews were conducted by phone or HIPAA-compliant Zoom with a purposive sample of patients with stage I–IV cancers, family caregivers, and oncology clinicians recruited from a large academic NCI-designated comprehensive cancer center in the southeastern US between January 2025 and July 2025. Open-ended questions prompted participants to discuss and elaborate upon what roles caregivers play in helping patients manage symptoms.

The COnsolidated criteria for REporting Qualitative research (COREQ) guidelines and checklist [16] were adhered to in the development of this report. This study was approved by the University of Alabama at Birmingham Institutional Review Board and performed in accordance with the ethical standards of the 1964 Declaration of Helsinki. Clinicians participants provided verbal informed consent and caregiver and patient participants provided signed informed consent prior interviewd and were given an incentive of $50 in the form of a pre-loaded Greenphire Visa gift card.

### Eligibility and recruitment

Family caregivers were eligible if they were 18 years of age or older, self-endorsed or were identified by the patient as “an unpaid relative or friend who knows them well and who provides regular support due to their cancer”, caring for a patient with stage I–IV cancer receiving active treatment, and English-speaking. Caregivers were excluded if they self-reported severe, untreated mental illness (e.g., schizophrenia, bipolar disorder, or major depressive disorder), dementia, active suicidal ideation, uncorrected hearing loss, or active substance abuse. Patients were eligible if they met the following inclusion criteria: 18 years of age or older; diagnosed with a stage I–IV cancer, including cancers of the bladder/kidney, bone/muscle, brain/spinal cord, breast, colon/rectum, endocrine, gynecologic, head/neck, leukemia or non-Hodgkin lymphoma, liver, lung, melanoma, pancreas, and prostate; and receiving active treatment (i.e., chemotherapy, targeted therapy, or immunotherapy). Patients were excluded if there was medical record documentation of active severe mental illness (e.g., schizophrenia, bipolar disorder, or major depressive disorder), dementia, active suicidal ideation, uncorrected hearing loss, or active substance abuse. Oncology clinicians, including (but not limited to) physicians, physician assistants, nurse practitioners, nurses, social workers, psychologists, behavioral health counselors, navigators, and administrators, had to have at least 1 year of experience working with patients with cancer and their caregivers. A targeted sample size of 12–25 individuals (per constituency) was targeted to achieve saturation based on qualitative guidance [17, 18].

Participants were recruited using purposive sampling to capture a range of cancer types (caregivers and patients) and professional disciplines and roles (clinicians). A clinical research coordinator (EO), working with clinic nurses, approached patients undergoing active cancer treatment and caregivers, using a list of patients recently enrolled in the cancer center’s electronic patient-reported outcome (ePRO) symptom monitoring program [19]. Clinicians were identified based on their involvement with the cancer center’s ePRO symptom monitoring program and invited by e-mail to participate in interviews.

### Interviews and data collection

One-on-one interviews were conducted by trained research staff between January 2025 and July 2025 using a semi-structured interview guide (available upon request) and prompts adapted from the team’s prior qualitative work [20–22]. After acquiring consent, digitally recorded interviews began with open-ended questions asking caregiver and patient participants about their background and experiences with cancer since diagnosis and their general experiences with treatment and symptoms to date. Interviews with clinicians started off by having them briefly describe their role and their patient population. Participants were then asked about how family caregivers assist patients with managing their symptoms, including specific roles and challenges. Follow-up prompts were used to have participants clarify and elaborate upon responses. Participant demographic characteristics were collected prior to the interview through structured, close-ended prompts.

### Analysis

Digitally recorded interviews were transcribed by a professional transcription company (Landmark Associates, Inc.) and subsequently uploaded into NVivo 14 software. Transcriptions were first reviewed for accuracy and immersion and all identifying information was removed. A thematic analysis approach then ensued for analysis following the approach outlined by Braun and Clark [23]. Line-by-line coding using open coding [24] was undertaken by the principal investigator (JNO), a board-certified hospice and palliative care advanced practice nurse and experienced family caregiver and qualitative researcher. Although prior research has described caregiver roles in cancer symptom management, an inductive open coding approach was chosen to allow for the emergence of novel insights and to avoid constraining analysis to existing frameworks and given the inclusion of oncology clinician perspectives alongside those of caregivers and patients. Using Miles, Huberman, and Saldana’s within and across case matrices strategy [25] to facilitate comparison of coded raw data, initial preliminary themes were formulated to address the study objective. Preliminary themes with descriptive definitions and all instances of raw text support from participants was critically reviewed by two members of the study team (RB, MS) to assess “fit” of the themes with the raw data. After refinements to the themes based on feedback, a second draft of the preliminary themes with supporting quotes were then circulated to all other members of the study team to ensure the collection of themes and raw data represented a complete picture of the data from all participants.

## Results

### Participant characteristics

We interviewed 20 family caregivers, 20 patients, and 25 oncology clinicians (Table 1). Caregivers averaged 59.2 years, were 60% White and 40% Black race, and were mostly female (70%) and the patient’s spouse/partner (40%). Nearly half of the caregiver participants (45%) provided support every day of the week, and half (50%) reported providing care 5 or more hours a day. Patients averaged 57.9 years and were mostly female (75%) with a wide range of cancer types and stages. Clinician participants averaged 37 years, were mostly female (84%), and included nurses (32%), physicians (24%), and non-clinical navigators (20%).

**Table 1:** Participant characteristics

	Family caregivers (*n* = 20)		Patients (*n* = 20)		Clinicians (*n* = 25)	
Age, mean (SD), range	Mean = 59.2; range = 34–81		Mean = 57.9; range = 34–80		Mean = 37.3; range = 23–53	
Gender						
Female	14	70%	15	75%	21	84%
Male	6	30%	5	25%	4	16%
Race						
White	12	60%	12	60%	15	60%
African American/Black	8	40%	8	40%	6	24%
Asian	0	0%	0	0%	4	16%
Employment						
Full/part time	12	60%	10	50%	n/a	n/a
Retired	6	30%	8	40%	n/a	n/a
Student	0	0%	0	0%	n/a	n/a
Homemaker	0	0%	0	0%	n/a	n/a
Unemployed	2	10%	2	10%	n/a	n/a
Marital status						
Married	12	60%	14	70%	n/a	n/a
Living with another	1	5%	1	5%	n/a	n/a
Single	2	10%	3	15%	n/a	n/a
Separated	0	0%	0	0%	n/a	n/a
Divorced	4	20%	1	5%	n/a	n/a
Widowed	1	5%	1	5%	n/a	n/a
Hispanic/Latino/a						
Yes	0	0%	1	5%	0	0%
No	20	100%	19	95%	25	100%
Highest education level						
8th grade or less	0	0%	0	0%	n/a	n/a
Some high school	2	10%	6	30%	n/a	n/a
Associates degree or vocational/technical school	2	10%	2	10%	n/a	n/a
Some college	8	40%	4	20%	n/a	n/a
College graduate	5	25%	1	5%	n/a	n/a
Master’s	1	5%	6	30%	n/a	n/a
Doctoral degree	2	10%	1	5%	n/a	n/a
Religious affiliation						
Protestant	6	30%	9	45%	n/a	n/a
No religious affiliation	2	10%	2	10%	n/a	n/a
Other	12	60%	9	45%	n/a	n/a
Location						
Urban	3	15%	2	10%	n/a	n/a
Rural	8	40%	9	45%	n/a	n/a
Suburban	9	45%	9	45%	n/a	n/a
What type of cancer do you have/does your care recipient have?						
Bladder and/or kidney	1	5%	2	10%	n/a	n/a
Breast	3	15%	5	25%	n/a	n/a
Colon and/or rectal	1	5%	0	0%	n/a	n/a
Head and neck	0	0%	0	0%	n/a	n/a
Leukemia and/or NHL	2	10%	1	5%	n/a	n/a
Lung	1	5%	3	15%	n/a	n/a
Ovarian	3	15%	2	10%	n/a	n/a
Pancreatic	3	15%	2	10%	n/a	n/a
Prostate	0	0%	0	0%	n/a	n/a
Other	6	30%	5	25%	n/a	n/a
What stage of cancer do you have/does your care recipient have?						
I	3	15%	4	20%	n/a	n/a
II	5	25%	5	25%	n/a	n/a
III	4	20%	2	10%	n/a	n/a
IV (including hematological cancers)	8	40%	9	45%	n/a	n/a
What is the relationship to the person you are caring for? This person is my…						
Husband or wife	8	40%	n/a	n/a	n/a	n/a
Partner	0	0%	n/a	n/a	n/a	n/a
Son or daughter	1	5%	n/a	n/a	n/a	n/a
Sister or brother	3	15%	n/a	n/a	n/a	n/a
Friend	1	5%	n/a	n/a	n/a	n/a
Other	7	35%	n/a	n/a	n/a	n/a
Days/week providing care						
1 day or less	2	10%	n/a	n/a	n/a	n/a
2–3 days/week	1	5%	n/a	n/a	n/a	n/a
4–5 days/week	4	20%	n/a	n/a	n/a	n/a
6 days/week	4	20%	n/a	n/a	n/a	n/a
Every day	9	45%	n/a	n/a	n/a	n/a
Hours/day providing care						
< 1 h/day	2	10%	n/a	n/a	n/a	n/a
1–2 h/day	5	25%	n/a	n/a	n/a	n/a
3–4 h/day	3	15%	n/a	n/a	n/a	n/a
5–6 h/day	2	10%	n/a	n/a	n/a	n/a
7–8 h/day	4	20%	n/a	n/a	n/a	n/a
> 8 h/day	4	20%	n/a	n/a	n/a	n/a
Clinical role						
Physician	n/a	n/a	n/a	n/a	6	24%
Nurse	n/a	n/a	n/a	n/a	8	32%
Lay navigator	n/a	n/a	n/a	n/a	5	20%
Other	n/a	n/a	n/a	n/a	6	24%

### Themes

As detailed below, analysis resulted in four main themes that described roles played by caregivers when assisting patients with managing symptoms and one cross-cutting theme that highlights a key difference and unique perception of the dynamic between caregivers and patients. Additional quotes for each theme are shown in Table 2.

**Table 2:** Key themes and exemplar quotes

Theme 1: Caregiving role—hands-on symptom management and functional compensation	We got one of those pill boxes for keeping up with day and night pills. And I came to the realization that I needed to be more involved in that. Even though instructions were given to it, they were written on the outside of the bottle, it still was confusing for her to keep up with what needed to be taken when. (CG9) I’ve gone through…helping him get a bath, helping him get out of his clothes, put his clothes back on…I’ve helped to feed him. And then there have been some instances where he wasn’t able to eat, but I was there spoon in hand, putting to his mouth just like a child. (CG3) He had to take care of me…he had to bathe me. He had to take me back and forth to the bathroom and help me get up and down. I mean, it was a full, taking care of somebody, like I was useless. I couldn’t eat anything…he cooked, he cleaned. He literally did everything that a CNA would do in a nursing home. (PT28) There are certain symptoms that we give them specific education on nausea or diarrhea, constipation, so they can help co-manage. (CLIN11)
Theme 2: Caregiving role—emotional support and motivation	In the beginning, he was kind of depressed, and so I spent a lot of time with him just trying to talk and encourage him and just trying to get his hope back up. (CG8) …just being there as someone to talk to because cancer’s hard and it’s bad sometimes. So having them as a sounding board or depression, a symptom like depression and things like that, that’s very helpful. (PT30) I think family plays a very integral role in terms of providing emotional support. (CLIN15)
Theme 3: Caregiving role—promotion of a symptom mitigating lifestyle	And of course with her cancer, she that high risk for bleeding…So that fall prevention is super important…[I got her] a side table at the head of her bed where she gets out of bed, so she automatically has a place she can put her hands. She doesn’t have to jump out onto the floor. (CG14) He lets me do my thing and sometimes he’ll say, just don’t push it, just slow it down. (PT37) My husband does all the pick up and drop off for our five-year-old because now my immune system is so low. I don’t really leave the house very often, but he does all of the activities for our son… (PT30)
Theme 4: Caregiving role—symptom reporter, advocate, and information seeker	I be on Google and be researching different stuff that can help her with the nausea. (CG6) I have to help listen during her appointments and…write notes down and keep up when her appointments are, because you have to write it down right then because…She just forgetting, not remembering things. (CG4) My husband … takes me to all the appointments. He sits with me through all my chemotherapy treatments, anything that’s medically related. (PT5) A lot of our family members will call on behalf of our patients. Some of our patients were too sick to even pick up the phone to call. I noticed that a lot of family members will also advocate for their family members, and I think that that’s kind of been the biggest thing. …So I think that that’s kind of been my biggest experience is that they just mostly advocate for their loved ones as far as making sure that they’re cared for. (CLIN23)
Theme 5: The challenge of finding the right balance between caregivers providing assistance while promoting and respecting patient independence and autonomy	the person that has cancer…they’re like, I don’t want to bother you…And it’s like, you’re not bothering me. Number one, don’t lie to me. Tell me what you need when you need it, or when you think you’re feeling a certain way, I got to know that in order to pre-plan to know what we got to do. (CG5) …she [the patient] doesn’t say what’s going on with her all the time…So that’s very challenging for me. If we have an appointment three days from now and we go to the doctor, oh yeah, I’ve been nauseous. Well, why didn’t you tell me we have two different nausea medicines here. Why wouldn’t you say something? So the first time that happened and the only time it happened, I just decided, well, you know what? I just give her a nausea pill every morning. (CG2) So he might've got ill with me a couple of times because maybe he felt like I should or could do more, but we just trod on through it. He knew he had to do it, I couldn't, and he did it. (PT28)

#### Theme 1: Caregiving role—hands-on symptom management and functional compensation

Many caregivers spoke about taking on direct responsibility for managing symptoms and medications and assisting patients with their day-to-day activities due to symptom-related limitations in their physical function. Patients affirmed this role, often crediting caregivers with helping them cope with symptoms and organize and take medications: “My husband…is on top of all my medications. He keeps track of the time and all that stuff. He does all that” (patient [PT] 30). Caregivers could also be observant of changes in the patient’s physical condition, requiring action: “You could tell when she stood [her skin] was getting plump…wasn’t staying up…It was getting more relaxed…I was like, oh my gosh, you really are dehydrated” (caregiver [CG] 12). Caregivers also provided hands-on support to assist patients due to the functional limitations imposed by physically limiting symptoms, including assistance with bathing, dressing, cooking, housework, and transportation, as this quote describes: “[I had to] drive him everywhere he goes…He could not grip the steering wheel. He had such swelling in his legs…His feet were getting numb…you can’t push a gas pedal or make a break if you’re [experiencing that]” (CG16). Out of 25 clinician interviews, only seven mentioned the role that caregivers play in direct symptom management, with most of these comments centered on medication management.

#### Theme 2: Caregiving role—emotional support and motivation

Both caregivers and patients highlighted how caregivers were a source of emotional support, encouragement, and motivation, particularly in the context of a situation that can be very depressing and demoralizing. As one caregiver stated: “There’s depression that comes with a diagnosis of cancer… And so I had to be her support while she was going through radiation and chemo…try to cheer her up as best I can and to look to the positive, which was the primary thing is not to feel as though you’re doomed” (CG11). Patients often described how this emotional support was closely intertwined with how they coped with psychological and physical symptoms, including pain, fatigue, and depression. Four of the 26 clinicians made comments relating to this emotional support role, which was acknowledged as an underappreciated aspect by one of these clinician participants: “So one of the biggest symptoms I’ve seen in our oncology patients that probably isn’t talked about as much is the psychological effects that it has on them. So I feel like the family members are able to…provide them some…emotional support” (CLIN24).

#### Theme 3: Caregiving role—promotion of a symptom mitigating lifestyle

Many caregivers talked about efforts to shape their care recipients’ daily routines in a way that prevented or eased symptoms. This might include preparing special diets, keeping patients hydrated, encouraging movement and rest, and making environmental modifications. As exemplified by one caregiver: “I’ve been juicing. And I tell her what she got to do is juice so she can have certain vitamins and stuff and to build her up…to keep from getting sick” (CG6). Another caregiver stressed the importance of getting more active: “I took her walking yesterday…at that international market and just walked around…she was hanging on to the buggy and she’s like, I feel good. I feel better.” Patients frequently acknowledged their caregiver engaging in these lifestyle efforts to help them with their symptoms: “She managed my diet, helped me manage my diet and stuff. I try to eat the things that I don’t think would upset me, but a lot of food you eat makes you nauseous” (PT31). Only a few clinicians briefly mentioned this aspect of the caregiving role, noting they “help with meals” (CLIN15) and “make sure they have food” (CLIN23).

#### Theme 4: Caregiving role—symptom reporter, advocate, and information seeker

Caregivers frequently served as symptom reporters, advocates, and information seekers, often by communicating with the oncology team and other services (e.g., pharmacies), finding information about managing symptoms, providing transportation and attending medical appointments with patients, and triggering decisions to seek additional help for worsening or new symptoms. Patients often saw caregivers as essential allies in this domain, especially when they felt too ill or fatigued to speak up themselves. As one patient participant eloquently summed up: “My partner has been my biggest advocate by far…the more you get into your treatments, the more you need somebody else’s voice alongside yours” (PT33). This was the area clinicians most often discussed when describing caregiver involvement in symptom management. They commonly noted that caregivers were essential in providing transportation to patients and were valuable sources of symptom information, often “escalating their concerns to the care team” (CLIN11), yet occasionally noting information overload. As discussed by one clinician: “They’re the advocate for the patient. I have a lot of family members who will tell me things that the patient won’t…The patient either doesn’t want to say it or they think it’s not that bad, and then we actually find that they’re having pretty serious symptoms” (CLIN21).

#### Theme 5: Challenges balancing assistance with respecting patient independence and autonomy

Caregivers spoke about a fundamental challenge that undergirded their role in supporting patients with their symptoms concerning knowing when and how much to step in and when let their care recipients take the lead: “I think my challenge for me personally is to make sure that I’m more compassionate and understanding and that I don’t make my own way, the way that it has to be done or should be done…I have to be very careful to try to remind myself, you’re not going through this. You don’t know what she’s going through and you just need to support her” (CG1). Patients also discussed this competing need between needing support but wanting autonomy: “What makes it difficult for a caregiver to help? Well, probably not knowing what to do because I do still feel good and can push myself and do the things I need to do…So it’s probably hard for a caregiver to know exactly how to help somebody like me who I’m not likely to ask for help unless I just can’t do it” (PT4). Patients were also sensitive to not wanting to ask for so much assistance that it overburdened their family members, adding an additional factor to the tension: “I know that it’s hard on them, but they don’t really admit it. I mean, their life changes as well as the patients” (PT29).

## Discussion

Across 20 caregivers, 20 patients with stage I–IV cancers, and 25 oncology clinicians, we identified five interrelated themes describing how caregivers help their care recipients manage symptoms, highlighting similarities and differences in what emerged across different participant groups. Caregivers were noted in interviews to assume a range of roles in symptom management, including assisting with direct hands-on support, providing emotional support and motivation, promoting preventative self-care and restorative behaviors, and serving as symptom reporters, advocates, and information seekers. Concerning these roles, a perceptual gap was apparent, namely that caregivers and patients described caregivers as central actors in the day-to-day management of symptoms, whereas many oncology clinicians tended to mostly focus on the role caregivers play as symptom reporters and advocates. Hence, efforts to support caregivers should not only equip them with skills and resources but also raise clinicians’ awareness of caregiver involvement to foster more coordinated, caregiver-inclusive approaches to symptom management in cancer.

Our findings that cancer caregivers provide hands-on symptom support including managing medications, emotional support and motivation, and serve as symptom reporters, advocates, and information seekers are consistent with prior reports by others [6, 9, 26, 27]. However, as noted in a 2018 systematic review by Ullgren and colleagues [9], the number of studies that specifically focus on the symptom management role of cancer family caregivers is comparatively small, underscoring the need for more attention to caregivers in this area. In our study, cancer caregivers reported assuming medication administration and symptom assessment and monitoring tasks in the home that would typically be performed by an oncology nurse in the hospital setting. This is concerning given national data suggesting that over 4 in 10 cancer caregivers do these medical/nursing tasks without any prior training [6], perhaps providing an additional possible explanation as to why the most prevalent reasons for CMS-designated avoidable emergency department visits for patients with cancer are all related to mismanaged symptoms, including pain, fever, nausea, and emesis [28]. Hence, it is imperative for oncology clinicians to become more familiar with the multi-dimensional role caregivers play in symptom support to patients in order to assess this dynamic during clinical encounters and provide appropriate guidance and education before symptom issues arise.

An under-recognized aspect of the caregiving role seen in our interviews compared to prior work was the efforts caregivers put towards preventative activities and tasks that minimized the future worsening or new occurrence of symptoms. Caregivers discussed preparing food and special diets to lessen nausea, making modifications to the living environment to prevent injury, organizing social interactions to avoid the risk of infection, and encouraging hydration, movement, and physical activity to improve energy levels and mood. Recent studies in the cancer context strongly validate the mitigating effects of some of these activities on symptoms, such as the positive impact of aerobic exercise on cancer-related fatigue [29] and special diets and protein intake on weight loss, fatigue, and nausea [30, 31]. These findings underscore the need to further explore and broaden the paradigm of symptom management in cancer care from a predominantly reactive approach to a proactive one that supports health promotion activities and mitigation strategies that lessen the emergence of new and worsening symptoms. Furthermore, the findings highlight the potential value of conceptualizing symptom management as a shared, dyadic process and suggest the need for interventions that support relational dynamics, such as select work by ourselves [32, 33] and others [34], rather than focusing solely on patients or caregivers in isolation.

Striking the right balance between offering support and assistance to patients while preserving and promoting their independence and autonomy was a key challenge for caregivers and patients to navigate, which has been noted in similar prior descriptive work [26]. Beyond the objective of achieving optimal health outcomes for the patients, caregivers wanted to provide enough support so that they felt useful and so patients did not feel uncared for or neglected. At the same time, not so much that patients felt helpless, irritated, and rebellious. Stemming from this, an important question going forward is whether providing too much assistance to patients contributes to deconditioning, strained caregiver-patient relationships, and ultimately worse symptoms? Borrowing insights from social support effectiveness theory [35, 36], clinicians and researchers might consider approaches to supporting caregivers symptom support role that stresses not only competence and skill but also the right type of support that is neither too much nor too little and which does not cause physical, psychological, or relational harm.

Several limitations should be noted for this study. First, the sample was purposive and recruited from a single cancer center and thus may not represent views generalizable to the entire population of family caregivers, patients with cancer, and oncology clinicians. However, a large proportion of participants identified as a racial minority, and the sample also included participants with diverse educational backgrounds. Together, these characteristics strengthen the study by capturing caregiving experiences shaped by social, cultural, and structural contexts and variation in health literacy and the capacity to understand and act on symptom-related information [37]. Second, this study focused on a wide range of cancer types and stages which may overlook important nuances to the caregiver symptom support role for particular cancers and at different stages. Given this, we recommend future efforts to develop support for caregivers incorporate as much cancer- and stage-specific content as is feasible and appropriate to the context.

 In conclusion, this study highlights the multifaceted roles that family caregivers undertake to support patients with cancer in managing symptoms. These roles extend well beyond physical assistance to include emotional support, proactive symptom prevention, and critical communication with healthcare teams. These findings suggest a pressing need to broaden the prevailing paradigm of cancer symptom management to formally recognize and integrate the proactive, health-promoting contributions of caregivers. As home-based cancer care becomes more prevalent and complex, the need to support caregivers in this role is growing. Future interventions and clinical strategies should not only educate and equip caregivers but also strengthen clinician awareness and engagement with caregivers, ensuring a more collaborative and anticipatory approach to symptom management in cancer care.

## Data Availability

Data available from the corresponding author upon reasonable request.
